# The association of different parenting styles among depressed parents and their offspring’s depression and anxiety: a cross-sectional study

**DOI:** 10.1186/s12888-021-03512-8

**Published:** 2021-10-09

**Authors:** Yanrong Wang, Honglan Shi, Yuan Wang, Xuan Zhang, Juan Wang, Yaoyao Sun, Jianwen Wang, Jiwei Sun, Fenglin Cao

**Affiliations:** 1 School of Nursing and Rehabilitation, Shandong University, No. 44 Wenhua Xi Road, Jinan, 250012 Shandong Province China; 2 Ningxia Medical University, No. 1106 South Shengli Street, Yinchuan, 750004 Ningxia China; 3 Mental Health Center, General Hospital of Ningxia Medical University, No. 804 South Shengli Street, Yinchuan, 750004 Ningxia China

**Keywords:** Child anxiety, Child depression, Depressed parent, Parenting styles, Latent profile analysis (LPA)

## Abstract

**Background:**

Parenting styles play a critical role in children’s development, especially for those in families with a depressed parent. To date, no study has explored whether youth perceptions of parenting style are heterogeneous in families with a depressed parent or whether heterogeneous parenting styles are associated with children’s internalizing symptoms.

**Methods:**

Participants were children aged 8–16 years who had a parent with major depressive disorder; they were enrolled through their parents, who were outpatients at two hospitals in Ningxia. Parenting styles were measured using the Parental Bonding Instrument. Youth depression and anxiety were measured using the Depression Self-Rating Scale for Children and the Screen for Child Anxiety-Related Emotional Disorders, respectively. We applied latent profile analysis to identify the subtypes of parenting styles with similar patterns. Differences between subtypes in relation to demographic variables and parenting style scores were calculated using one-way ANOVAs, Wilcoxon rank sum tests, and chi-squared tests. Bivariate logistic analyses were conducted to examine the associations between parental bonding subtypes and children’s depression and anxiety.

**Results:**

Four parenting styles were identified through latent profile analysis: care-autonomy, overprotection-indifference, indifference, and undifferentiated parenting. Youth with care-autonomy parents had a lower risk of depression (OR: 0.16; 95% CI: 0.06–0.41) and anxiety (OR: 0.22; 95% CI: 0.10–0.48), while indifference parenting increased children’s risk of depression (OR: 5.29; 95% CI: 1.30–21.54) more than undifferentiated parenting.

**Conclusions:**

Children with a depressed parent had heterogeneous perceptions of parenting styles. Mothers’ and fathers’ parenting styles were largely congruent. Care-autonomy parenting (high care and high autonomy) may decrease children’s risk of depression, whereas indifference parenting (low care and autonomy) may increase their risk of depression.

**Supplementary Information:**

The online version contains supplementary material available at 10.1186/s12888-021-03512-8.

## Introduction

Substantial evidence shows that children and adolescents of parents with depression have an elevated risk of internalizing symptoms (e.g., depression and anxiety) [1–3]. Previous studies also found that children of parents with depression histories show maladaptive ways of self-regulating sadness [4], more conduct problems [5], and frequent experiences of bullying by peers [6]. There are several potential mechanisms to explain the associations between parental depression and offspring’s depression and anxiety. One of the most important factors is genetics, which has been well documented in previous studies [7, 8]. Additionally, prenatal exposures and experiences, parenting, exposure of offspring to childhood adversities, and children’s vulnerabilities are all reported to be related to the intergenerational transmission of depression [9]. Parents’ attitudes and behaviors in day-to-day interactions with their offspring, that is, their parenting style, play a critical and essential role in children’s neuropsychological development [10]. However, depressed parents are more likely to exhibit both withdrawn and intrusive behaviors than parents who have not experienced depression, and these behaviors are related to greater symptoms of internalizing psychopathology in young people [11–16]. To explore associations between parenting style and children’s depression/anxiety among depressed parents is of great significance for improving family environment and preventing the risk of offspring’s depression and anxiety.

In classic studies focusing on parenting styles, Baumrind [17, 18] defined a tripartite model based on the interaction between affection, communication, and control, yielding three parenting styles: authoritative, authoritarian, and permissive. Maccoby and Martin [19] revised Baumrind’s conceptual framework and defined four parenting styles: authoritative (high warmth and high strictness), authoritarian (low warmth and high strictness), indulgent (high warmth and low strictness), and neglectful (low warmth and low strictness); this framework distinguishes between the variation in warmth among low strictness categories. Schaefer utilized factor analysis and identified three factors based on children’s reports about their parents’ behaviors, which were interpreted as acceptance vs. rejection, psychological autonomy vs. psychological control, and firm control vs. lax control [20]. Parker proposed a two-domain structure of perceived parenting styles using care and overprotection [21], which was then developed into a multifactor structure under different cultural contexts. Additional parenting styles, such as “unlabeled” [22], “controlling-indulgent” [23], “neglectful/punishing” [24], “easygoing,” and “tiger” [25] were also reported in previous studies. However, there are some theoretical and empirical weakness that need to be improved.

On the one hand, the number of parenting subtypes has been predetermined by using arbitrary cut-off scores on dimensions to create parenting style groups (e.g., 1 standard deviation above or below the means on the relevant dimensions), leading some potential parenting subtypes to be omitted [26]. Although the previously used variable-centered approaches (i.e., factor analysis and principal component analysis) are helpful in understanding the relative contribution of a specific characteristic to an outcome, a person-centered approach (i.e., latent profile analysis [LPA]) can provide a more nuanced understanding of a phenomenon by including multiple parenting dimensions and identifying unique profiles within a large heterogeneous population. Furthermore, the optimal number of groups is determined by an overall consideration of theoretical conceptualization, model fit indices, and validity evidence, which are more valid and reliable [27]. Several studies have investigated naturally occurring parenting styles using person-centered approaches in western samples [22–24] and a Chinese-American sample [25]. Zhang and colleagues [26] used LPA to identify four parenting style subtypes among early adolescents in healthy families in China: authoritative, authoritarian, average-level undifferentiated, and high-level undifferentiated. However, no study has used LPA to explore parenting styles in families with depressed parents. A review found that parents with depression were less sensitive to their children’s needs and had maladaptive parenting behavior, which are negatively correlated with children’s depression and other psychological difficulties [14]. However, they have not explored whether parenting styles are heterogeneous in families with depressed parents or whether such heterogeneity will lead to different outcomes for children.

On the other hand, parenting styles differ across different cultures, and the best parenting methods identified in studies conducted in western countries are not suitable for families in China. According to Baumrind’s parenting styles, in America, authoritative parents who are highly demanding and responsive are considered remarkably successful in protecting their offspring from behavioral problems [28]. However, benefits of authoritarian parenting have been found in Chinese-American [29] and African-American families [30] in cross-cultural studies. Additionally, recent studies have identified benefits related to indulgent parenting, that is, parental warmth without strictness [31, 32]. Larger cultural differences may exist in east Asian countries than in western countries. In the context of Confucianism, children in east Asian countries tend to consider strictness as normal parenting behavior and consider obeying their parents and not fighting with them as an external display of respect. Therefore, children in China may have different perceptions of the same parenting styles and different neuropsychological development compared with children raised in western cultures.

Parenting style quality has been reported to be associated with the child’s later mental health [14, 33]. Depression in high-risk children and adolescents is being researched more often and has been gaining clinical attention over the years, given the three-fold increase in such children’s rate of depression, compared with offspring of parents without depression [34]. Thus, it would be useful to investigate the relationship between parental bonding and various clinical outcomes among children and adolescents at high-risk for depression. The main purpose of the current study was to explore the children’s perceived parenting subtypes of depressed parents using LPA and to further explore the association between parental subtypes and children’s depression and anxiety.

## Methods

### Participants

Study participants were children aged 8–16 years who were part of an ongoing project to foster mental resilience in the offspring of parents with depression. Offspring of parents with major depressive disorder were enrolled through affected parents receiving outpatient treatment at two of the authors’ local hospital. Clinicians systematically enquired if patients with major depressive disorder had biological children in the eligible age range. Only one child from each family participated in the research. For families who had more than one child aged 8–16 years, only the elder one was included. All selected children lived with the affected parent. We excluded parents with psychosis, bipolar disorder, mania, hypomania, and those who were divorced, separated, single, and remarried.

Participants completed all measures via telephone or face-to-face surveys, which were conducted by research staff who received rigorous training prior to this fieldwork. The study conformed to the 1964 Helsinki declaration and its later amendments or comparable ethical standards and was approved by the Ethics Review Board of the first author’s university. Written informed consent was obtained from the parents, and oral consent was obtained from the children before the interview.

Of a total of 273 children who met the inclusion criteria, 28 were excluded because of incomplete data or unwillingness to join the research. Data were collected from 245 (89.7%) participants from November 2018 to November 2020. The mean age of participants was 12.19 (SD: 2.78) years, and 51.4% were girls. Children whose families consented to participate did not differ from those who did not consent in terms of gender or age characteristics (p<0.05).

### Measures

#### Parental bonding instrument (PBI)

The Parental Bonding Instrument (PBI) is a self-report questionnaire developed to measure the subjective experience of being parented up to the age of 16 years [35]. Individuals were asked to answer the PBI based on parent-child interactions before the age of 16. It is the most widely used measure of parenting style in a range of clinical and non-clinical participant groups [36]. The PBI contains 25 items with responses on a four-point Likert-type scale ranging from 0 (very unlikely) to 3 (very likely). Individual scores were obtained by averaging the item scores for each dimension, with higher scores indicating higher levels of the particular parenting dimension. The PBI consists of four principal dimensions: care, indifference, overprotection, and autonomy [37]. In this context, “care” represents caring, warm, and loving parenting; “indifference” is perceived as both indifference or coldness and intrusiveness or aggression; “overprotection” indicates opposition to independence and assertiveness; and “autonomy” represents allowance of autonomy and independence. The PBI was suggested to have a four-factor structure in a Chinese sample [38]. The Chinese version of the PBI has demonstrated good reliability and validity [37, 38]. Cronbach’s alphas of the four subscales were 0.64, 0.68, 0.74, and 0.82 for mothers, and 0.63, 0.69, 0.77, and 0.84 for fathers.

#### Depression self-rating scale for children (DSRSC)

We used the Depression Self-Rating Scale for Children (DSRSC) [39] to measure the depression status of children in the past week. The DSRSC is a self-report questionnaire containing 18 items, with responses on a three-point scale (0 = never; 1 = sometimes; 2 = often). Higher scores indicate depression severity. In China, the psychometric properties of the DSRSC for ages 8–16 years have been found to be satisfactory, with good reliability and validity. The cut-off score for the DSRSC is 15, with more than 15 points indicating a depressive state [40].

#### The screen for child anxiety-related emotional disorders (SCARED)

The Screen for Child Anxiety-Related Emotional Disorders (SCARED) [41] aids psychologists in diagnoses, scientific research, and epidemiological investigations and was developed to assess anxiety disorders in children in the past 3 months. SCARED is a self-report questionnaire comprising 41 items scored on a three-point scale (0 = never, 1 = sometimes, 2 = often). Acceptable reliability and validity have been established for this scale in China for ages 8–16 years. The 80th percentile of the total score had the best sensitivity and specificity to identify potential anxiety disorder in previous study [42]. Additionally, the 80th percentile of SCARED in the current study was 23, with more than 15 points indicating an anxiety state.

#### Socio-demographic characteristics

Parents’ demographic characteristics included parental age, social economic status (SES), parents’ educational level, paternal or maternal depression, and depression duration. Children’s demographic characteristics included age, gender, only child (yes/no), smoking (yes/no), and drinking (yes/no). Measures of the socio-demographic characteristics are provided in the supplementary materials.

### Statistical analyses

Statistical analyses were conducted using SPSS (Statistical Package for the Social Sciences, Chicago, IL, USA) version 20.0 and Mplus 7.4 software (Statistical Innovation, Belmont, MA, USA). Children’s perceived paternal and maternal parenting styles were used to classify parenting subtypes using LPA. The Akaike information criterion (AIC), Bayesian information criterion (BIC) [43] and sample-size adjusted BIC (aBIC) were used to determine model fit, with lower values indicating better fit. The Lo-Mendell-Rubin likelihood ratio test and bootstrapped likelihood ratio test were used to compare model fit improvements between models with k and k-1 classes. Higher entropy values indicate more acceptable classification accuracy. Differences among parenting subtypes in relation to socio-demographic characteristics and PBI scores in four dimensions were compared using one-way ANOVA and chi-square tests. Post hoc tests were conducted with *Bonferroni* multiple-comparison correction. Since Parents’ depression duration does not conform to a normal distribution, the Kruskal-Wallis test was used to compare parents' depression duration of different parenting subtypes. To further explore the association between parenting subtypes and children’s depression and anxiety, bivariate logistic analyses were conducted. *P* < 0.05 was considered statistically significant.

## Results

### Identifying parenting subtypes using LPA

We conducted LPA models using the standardized scores of the parenting dimensions (see Table 1 for model fit statistics). Among the models, the four-profile model had smaller AIC (4983.342), BIC (5133.896), and aBIC (54,997.589), and higher entropy (0.854). Given the clinical implication that the four-class solution provided the most conceptually coherent description of the PBI, it was chosen as the most appropriate solution. A five-profile model was also considered (AIC = 4892.972, BIC = 5075.037, aBIC = 4910.201, entropy = 0.872), but the added fifth profile was too small to represent a meaningful subtype (2.04% of the sample).

**Table 1:** Goodness-of-Fit Indices of Latent Profile Models Comprising One to Five Classes (*N* = 245)

No. of profiles	AIC	BIC	aBIC	Entropy	LRT	BLRT	n(%)				
							Profile 1	Profile 2	Profile 3	Profile 4	Profile 5
1	5646.467	5702.487	5651.768								
2	5212.825	5300.35	5221.108	0.801	451.462*	− 2807.233***	131(53.46%)	114(46.54%)			
3	5080.305	5199.347	5091.570	0.829	150.520	− 2581.412***	52(21.22%)	123(50.20%)	70(28.57%)		
4	4983.342	5133.896	54,997.589	0.854	114.963	− 2506.152***	21(8.57%)	53(21.63%)	59(24.08%)	112(45.71%)	
5	4892.972	5075.037	4910.201	0.872	108.370	− 2448.671***	5(2.04%)	16(6.53%)	106(43.27%)	65(26.53%)	53(21.63%)

*AIC* Akaike information criterion, *BIC* Bayesian information criterion, *aBIC* adjusted Bayesian information criterion, *LRT* Lo-Mendell–Rubin likelihood ratio test, *BLRT* bootstrapped likelihood ratio test. *P < 0.05; ***P < 0.001

**Fig. 1:**
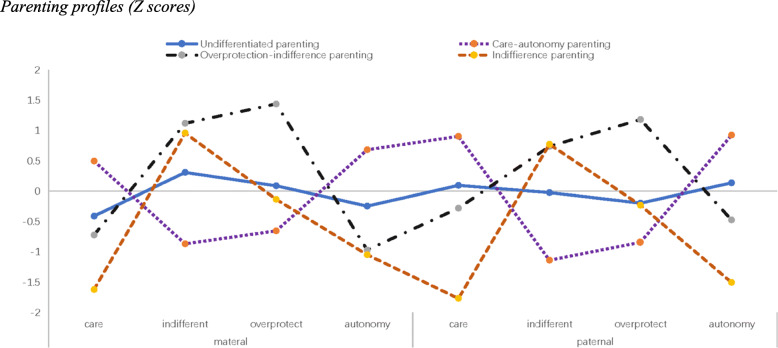
Parenting profiles (Z scores)

The four-profile models are shown in Fig. 1, and scores on the PBI are shown in Table 2. The first profile comprised 8.6% of the sample. Compared to the other profiles, this profile demonstrated high levels of care and autonomy and low levels of indifference and overprotection and was named “*care-autonomy parenting*.” The second profile comprised 21.6% of the sample and included children reporting their parents as having the highest levels of overprotection and indifference, and the lowest autonomy for mothers and lower levels of care and autonomy for fathers, which was named “*overprotection-indifference parenting*.” The third profile comprised 24.1% of the sample and had the highest levels of indifference, which was similar to the second profile, but lower levels of overprotection and the lowest levels of care and autonomy, and was named “*indifference parenting.*” The fourth profile comprised almost half of the sample (45.7%). There was no dimension on which the score was significantly higher or lower compared to the other dimensions within this group, and mean scores on the PBI among this profile were similar to the mean scores in the entire sample. Hence, this profile was named “*undifferentiated parenting.*”

**Table 2:** Descriptive scores of four parenting style characteristics (*N =* 245)

	1. Indifference	2. Care-autonomy	3. Overprotection-indifference	4. Undifferentiated	*F*	*P* value	Post hoc *tests*
Maternal parenting style							
Care	13.80 ± 4.11	19.71 ± 1.64	16.22 ± 2.40	17.12 ± 2.42	34.800	0.000	2 > 1 = 3
Indifferent	15.23 ± 4.32	8.41 ± 2.09	15.61 ± 2.87	12.88 ± 2.38	75.559	0.000	1 = 3 > 2; 3 > 4
Overprotection	13.80 ± 2.60	11.86 ± 2.76	19.81 ± 2.47	14.51 ± 2.20	108.926	0.000	3 > 1 > 2; 3 > 4 > 2
Authoritarian	13.80 ± 4.88	19.86 ± 2.60	14.06 ± 2.92	16.72 ± 2.50	44.022	0.000	2 > 1; 4 > 3 > 2
Paternal parenting style							
Care	10.38 ± 2.69	19.54 ± 1.69	15.61 ± 2.72	16.49 ± 2.12	86.709	0.000	2 > 3 = 4 > 1
Indifferent	16.52 ± 5.31	8.75 ± 2.16	16.16 ± 2.90	13.38 ± 2.39	74.769	0.000	1 = 3 > 2; 3 > 4 > 2
Overprotection	13.85 ± 2.70	11.54 ± 2.67	19.47 ± 2.85	14.04 ± 2.14	103.419	0.000	3 > 4 = 1 > 2
Authoritarian	9.47 ± 2.52	20.01 ± 2.61	14.25 ± 3.07	16.51 ± 2.73	84.405	0.000	2 > 4 > 3 > 1

Table 3 shows the socio-demographic characteristics of the four groups. There were no between-group differences except smoking and parent’s highest education. Children under *indifference parenting* had the highest rate of smoking (28.6%), and parents with *care-autonomy parenting* had the highest completion rate of college or above education (49.1%).

**Table 3:** Socio-demographic characteristics of four parenting profiles (*N =* 245)

	Overall sample (*N* = 254)	1. Indifference (*n* = 21)	2. Care-autonomy (*n* = 53)	3. Overprotection-indifference (*n* = 59)	4. Undifferentiated (*n* = 112)	*χ*^*2*^*/ F/Z*	*P* value
Age	12.19 ± 2.79	12.10 ± 2.83	11.89 ± 2.59	12.66 ± 2.79	12.11 ± 2.87	0.810	0.490
Gender						5.148	0.161
Boy	119 (48.6)	6 (28.6)	25 (47.2)	27 (45.8)	61 (54.5)		
Girl	126 (51.4)	15 (71.4)	28 (52.8)	32 (54.2)	51 (45.5)		
One child						3.426	0.331
One	102 (41.6)	11 (52.4)	25 (47.2)	26 (44.1)	40 (35.7)		
More than one	143 (58.4)	10 (47.6)	28 (52.8)	33 (55.9)	72 (64.3)		
Residential address						3.568	0.312
Urban	153 (62.4)	15 (71.4)	35 (66.0)	40 (67.8)	63 (56.3)		
Rural	92 (37.6)	6 (28.6)	18 (34.0)	19 (32.2)	49 (43.8)		
Socioeconomic Status						11.379	0.077
Above average	29 (11.8)	1 (4.8)	11 (20.8)	7 (11.9)	10 (8.9)		
Medium	174 (71.0)	18 (85.7)	38 (71.7)	41 (69.5)	77 (68.8)		
Below-average	42 (17.1)	2 (9.5)	4 (7.5)	11 (18.6)	25 (22.3)		
Substance use							
Smoking	22 (9.0)	6 (28.6) ^a^	0 (0) ^b^	8 (13.6) ^a^	8 (7.1) ^a^	17.067	0.003
Drinking	51 (20.8)	8 (38.1)	7 (13.2)	15 (25.4)	21 (18.8)	6.715	0.082
Parents’ depression						3.493	0.322
Father	75 (30.6)	4 (19.0)	16 (30.2)	23 (39.0)	32 (28.6)		
Mother	170 (69.4)	17 (81.0)	37 (69.8)	36 (61.0)	80 (71.4)		
Depressed Parents’ age	38.80 ± 4.68	39.29 ± 4.27	39.36 ± 4.47	39.25 ± 5.18	38.21 ± 4.56	1.098	0.351
Parents’ highest education						13.320	0.038
Primary school	115 (46.9)	6 (28.6)	20 (37.7)	28 (47.5)	61 (54.5)		
Junior high school	44 (18.0)	6 (28.6)	7 (13.2)	15 (25.4)	16 (14.3)		
College or above	86 (35.1)	9 (42.9)	26 (49.1)	16 (27.1)	35 (31.3)		
Parents’ depression duration		127.98	135.52	124.28	115.47	5.219	0.156
Children’s depression	123 (50.2)	18 (85.7) ^a^	10 (18.9) ^b^	39 (66.1) ^ac^	56 (50.0) ^c^	37.377	0.000
Children’s anxiety	140 (57.1)	16 (76.2) ^a^	17 (32.1) ^b^	37 (62.7) ^a^	70 (62.5) ^a^	18.770	0.000
Children’s depression	15.73 ± 10.03	24.67 ± 8.14 ^a^	8.24 ± 7.32 ^b^	19.97 ± 9.93 ^ac^	15.38 ± 9.00 ^c^	24.384	0.000
Children’s anxiety	31.67 ± 21.25	45.14 ± 22.09 ^a^	18.94 ± 16.69 ^b^	38.75 ± 23.14 ^ac^	31.45 ± 18.88 ^c^	13.001	0.000

Post hoc analyses show significant differences among groups: same letter indicates no difference

### Associations between parenting subtypes and Children’s depression and anxiety

More than half of the children from families with a depressed parent had depression (50.2%) and anxiety (57.1%). Children with *care-autonomy* parents exhibited the lowest rates of depression and anxiety-related problems, while those with *indifference* parents had the highest levels of depression and anxiety. In the multivariate logistic model, only the *indifference parenting* and *care-autonomy parenting* profiles remained significant after adjusting for confounders (Table 4). The odds of children experiencing *indifference parenting* being positive for depression were 5.29 times (95% CI: 1.30–21.54) greater than the odds of children experiencing *undifferentiated parenting* being positive for depression. However, the odds of children experiencing *care-autonomy parenting* being positive for depression and anxiety were only 0.16 times (95% CI: 0.06–0.41) and 0.22 times (95% CI: 0.10–0.48) less than the odds of children experiencing *undifferentiated parenting* being positive for depression and anxiety, respectively.

**Table 4:** Multivariate logistic regression models for associations between parenting style and youth depression and anxiety (*N* = 245)

	Depression		Anxiety	
	OR (95% CI)	Adjusted OR (95% CI)	OR (95% CI)	Adjusted OR (95% CI)
Indifference	6.00 (1.67–21.51) **	5.29(1.30–21.54) *	1.92 (0.65–5.62)	1.46 (0.45–4.77)
Care-autonomy	0.23 (0.10–0.50) ***	0.16 (0.06–0.41) ***	0.28 (0.14–0.56) ***	0.22(0.10–0.48) ***
Overprotection-indifference	1.95 (1.01–3.75) *	1.64 (0.78–3.45)	1.009 (0.52–1.93)	0.73 (0.36–1.50)
Undifferentiated	1	1	1	1

*P < 0.05; **P < 0.01; ***P < 0.001

Adjusting for Smoking, Parents’ highest education, Children’s depression, Children’s anxiety

## Discussion

The present study makes an important contribution to the literature on parenting styles. It is the first study to use LPA to examine the potential subtypes of both maternal and paternal parenting styles among children from families with a depressed parent.

The results showed that parenting style is heterogeneous in families with a depressed parent. Four parenting styles were identified: care-autonomy, overprotection-indifference, indifference, and undifferentiated. Children’s perceptions of mothers’ and fathers’ parenting were largely congruent; children with care-autonomy parents were at a lower risk of depression and anxiety, while indifference parenting increased children’s risk of depression, followed by undifferentiated parenting.

A novel contribution of our study was that we used LPA to identify patterns of parenting emerging from the ratings of high-risk children along four dimensions of PBI. This differs from past research regarding families with a depressed parent, which largely relied on comparing parenting styles between families with a depressed parent with families without one. Several previous studies [1, 14] have examined associations among mothers’ or fathers’ parenting styles and internalizing symptoms among children with depressed parents by differentiating parenting dimensions for either fathers or mothers. To our knowledge, this is the first study to include all parenting dimensions for both fathers and mothers by employing person-centered analyses.

Additionally, we found a high level of agreement across parenting characteristics of mothers and fathers, with all participants living with two parents classified in the same profile. Our results are comparable to those of Fletcher and Sellers [44], who found that 72% of two-parent families in their large U.S. sample had consistent parenting styles. Such congruence is not surprising given the likelihood of assortative mating, the possibility that parents influence each other’s beliefs and values, and, perhaps, informant effects (e.g., children rating both parents together).

Our results show that depressed parents’ parenting is not as monolithically negative as the literature suggests [12]. Four different parenting styles existed among Chinese depressed parents. Parents were most frequently classified as showing undifferentiated parenting, which provides moderate support, warmth, and love, along with moderate intrusiveness and restraint. The undifferentiated parenting styles were previously observed in Zhang et al.’s research [26] wherein healthy families were recruited. However, unlike Zhang et al.’s research, no high-level undifferentiated parenting style was identified in the present study. This indicated a potential poor parenting environment among depressed parents. In comparison with the classic parenting styles from Baumrind [45], only permissive and authoritarian styles were found. No authoritative parenting style, which was previously reported in healthy parents and generally considered the ideal style, was identified in the present study. Moreover, overprotection-indifference parenting and indifference parenting, which were generally considered undesirable parenting practices, were also identified in the current study. The above findings suggest that the parenting status among depressed parents is worrying. Parental depressive symptoms were correlated with negative family atmosphere and were predictive of children’s inattentive and hyperactive symptoms, depression, and anxiety [46, 47]. Our findings revealed the significance and urgency of providing parenting interventions to depressed parents.

Similar to previous studies on parenting styles [48, 49], we found that children under indifference parenting were more likely to be depressed. Compared with children in overprotection-indifference parenting families, those in indifference parenting families were extremely lacking in interactions with their parents. As Chinese children tend to be shy or reserved, indifference from parents has great potential to lead to children being bullied and further risk for depression and anxiety [50]. However, it is important to note that the sample size of the indifference parenting subtype was small and that the confidence intervals of the effect size were large; therefore, the result needs to be cautiously interpreted.

Overprotection-indifference parenting, which had higher level of overprotection but the same level of indifference as indifference parenting, was also identified in the current study. There are some differences between the two parenting styles. Overprotective-indifferent parents rarely appreciate or praise their children, while frequently using coercive discipline, have more control and restraint over their children, and rarely consider their children’s thoughts and wishes. Indifferent parents were found to have low levels of control and were largely unresponsive toward their children, consistent with previous studies [19, 51]. Compared to undifferentiated parenting, the overprotection-indifference parenting style was not found to be associated with depression. In contrast to our findings, some studies found that authoritarian parenting, which was less warm and highly strict, and similar to overprotection-indifference parenting, was significantly related to the presence of depression [52, 53]. Excessive protection may prevent a child’s psychological needs for autonomy and competence from being met [54, 55] and affect their overall social development and well-being, resulting in more severe symptomatology. However, in our study, overprotection did not have a negative effect, which is consistent with Liu’s study [38]. This may be because the overprotection factors on the PBI has demonstrated cross-cultural inconsistencies [26, 56]. An item such as “Tended to baby me,” classified as reflecting overprotection in one culture, may be interpreted as reflecting care in another [57]. Overprotection-indifference parenting showed low levels of autonomy. However, autonomy is not as deeply imbedded a construct in eastern cultures as it is in western ones. Lack of autonomy, therefore, may be viewed more neutrally by children in eastern cultures. In addition, Chinese parents were highly attached to their children compared with other cultural populations [38], leading some children to perceive the overprotection as normal. This could partially explain why overprotection or lack of autonomy was not viewed as a risk factor for children’s depression in our sample.

Compared to undifferentiated individuals, the care-autonomy parenting group had a lower incidence rate of depression and anxiety. Both mothers and fathers who were classified under care-autonomy parenting treated their children as independent individuals, had an accepting attitude toward the child, made few demands for mature behavior, and respected and understood their children, thus providing more empathy, warmth, and support. This style has been recognized as a suitable rearing combination in a previous study [56]. However, another previous study reported that permissive parenting (high warmth and low strictness, similar to care-autonomy parenting) tends to be related to negative developmental outcomes for children and adolescents [58]. This inconsistency may be because of the different analytic approaches. Previous variable-centered approaches usually utilized high percentile scores to define whether an individual belongs to one kind of parenting style, which may lead to extreme parenting scores. The permissive parenting style was once divided into indulgent (high warmth and low strictness) and neglectful (low warmth and low strictness) parenting [19]. However, a person-centered approach was conducted an average levels of parenting style and showed the positive characteristics of care and autonomy. Furthermore, the children in the current study were raised under China’s one-child policy and have been accustomed to enjoying care from the family. Furthermore, it is difficult for them to perceive this care as negative and indulgent.

The findings of this study have implications for depressed parents regarding modeling the ideal parenting style. Indifference parenting styles have harmful effects on children, whereas the care-autonomy parenting style benefits children. Depressed parents can heed this research knowledge, examine their own parenting styles, and possibly change their parenting styles to ensure the best outcomes for their children. To do so, clinicians can inform parents how each parenting style looks and guide parents to offer adequate caring, warmth, loving, and support for their children, while encouraging their independence. Second, these results have implications for family-oriented prevention strategies in that they provide information on which combinations of parenting dimensions are particularly relevant. Counselors and family therapists could also utilize the information detailed in each cluster for foster children from depressed families to help them learn more coping styles or resilient coping skills to reduce the risk of depression and anxiety.

### Limitations

This study has some limitations. First, its cross-sectional design limits our ability to infer the temporal and potential causal direction between parenting styles and children’s depression and anxiety, and it could not be ascertained whether parenting styles changed over time. Future longitudinal or cross-lagged designs can be used to provide support for causal mechanisms. Second, our study focused on the children’s reports, which may have introduced some bias. Fletcher [59] pointed to the extensive literature demonstrating that adolescents can provide accurate and reliable reports of their parents’ parenting practices, and some have claimed that because adolescents act on the basis of their perceptions, studies should focus on adolescents’ ratings. However, the children’s depression/anxiety may also influence the parenting they received and their perceptions of parenting styles. It would be worthwhile to include reports from both parents and children in future research to determine whether similar profiles emerge in their reports and to examine consistency between the reporters. Third, only depressed parents who were visiting clinics were recruited, and depressed parents not receiving treatment were not included; this may have led to selection bias. The small sample size of the present study limits the generalizability of our findings, which needs to be addressed. The small sample size in the “indifference cluster” led to large confidence intervals, and the impact of this cluster on children’s depression need to be cautiously interpreted. In addition, measurement bias and confounder bias were also non-negligible limitations. Finally, it is important to note that the pronatalist policy of China has changed since 2016, the year that the two-children policy was enacted. The children recruited in the current study, from 2018 to 2020, experienced various impacts under the two-children policy. Although the PBI measures children’s average perceptions of parenting style up to the age of 16 years, the potential bias caused by the change of policy cannot be ignored. Specifically, the three-children policy was published this year, and the transformation of the associations between parenting style and children’s risk of mental disorders need to be further studied and traced. Owing to the many limitations, future studies need to follow up participants longitudinally, include parents and children at the same time, increase sample representativeness and sample size, and include as many confounders as possible.

## Conclusion

The present study identified the potential types of parenting styles among children and adolescents from families with a depressed parent, using a potential profile analysis approach. The study presents three main findings. First, four types of parenting styles were identified among families with a depressed parent: care-autonomy, overprotection-indifference, indifference, and undifferentiated. Second, children with care-autonomy parents had lower risks of depression and anxiety, whereas those with indifferent parents had a higher risk of depression than undifferentiated parents. Third, mothers’ and fathers’ parenting styles were largely congruent.

## Supplementary Information

**Additional file 1.** Appendix 1. Covariate classification definition.

## Data Availability

The datasets used and analyzed during the current study are available from the corresponding author on reasonable request.

## Abbreviations

AIC: Akaike information criterion
aBIC: Sample-size adjusted BIC
BIC: Bayesian information criterion
DSRSC: Depression Self-Rating Scale for Children
LPA: Latent profile analysis
PBI: Parental Bonding Instrument
SCARED: Screen for Child Anxiety-Related Emotional Disorders
SES: Socioeconomic status
