# Supplementary material for: The association of different parenting styles among depressed parents and their offspring’s depression and anxiety: a cross-sectional study
Source: BMC Psychiatry. 2021 Oct 9;21:495. doi: 10.1186/s12888-021-03512-8 (PMC8502291; doi:10.1186/s12888-021-03512-8)
Supplement: Supplementary file 1 — Additional file 1. Appendix 1. Covariate classification definition. [file 12888_2021_3512_MOESM1_ESM.docx]

Appendix 1

Covariate classification definition

| Variable | Measure | Code |
| --- | --- | --- |
| Age | How old are you? | M±SD |
| Gender | Are you a boy or a girl? | boy = 0, girl = 1 |
| One child | Are you the only child in your family? | Only child = 0, more than one = 1 |
| Residential address | Do you live in an urban or in a rural area? | Urban = 0, rural = 1 |
| Socioeconomic status | Compared with your neighbors, how do you feel about your socioeconomic status? | better than those around them = 1  similar to those around them = 2  worse than those around them = 3 |
| Smoking | Have you ever tried smoking, even a few puffs? | yes = 0, no = 1 |
| Drinking | Have you ever drank alcohol (not just a small sip)? | yes = 0, no = 1 |
| Paternal or maternal depression  (Parent report) | Who is the depressed parent, the father or the mother? | paternal depression = 0, maternal depression = 1 |
| Depressed Parents’ age (Parent report) | How old are you? | M±SD |
| Parents’ educational level | What is the highest education level of your father and mother? | at least 1 parent completed primary school = 1  at least 1 parent completed high school = 2  at least 1 parent completed college = 3 |
| Children’s depression | Depression Self-Rating Scale for Children (DSRSC) score. | M±SD;  DSRSC＜15 = 0,DSRSC≥15 = 1 |
| Children’s anxiety | The Screen for Child Anxiety-Related Emotional Disorders (SCARED) score. | M±SD;  SCARED＜23 = 0, SCARED≥23 = 1 |
